# Response to ‘Letter to the Editor’: Blood flow restriction training attenuates changes in local muscle endurance: At odds with previous work?

**DOI:** 10.1113/EP092002

**Published:** 2024-06-28

**Authors:** Akito Ida, Kazushige Sasaki

**Affiliations:** ^1^ Department of Life Sciences, Graduate School of Arts and Sciences The University of Tokyo Tokyo Japan

**Keywords:** chronic adaptations, linear mixed model, occlusion, resistance exercise

1

We appreciate the opportunity to respond to the comments and criticisms made by Hammert et al. (2024) on our recent paper (Ida & Sasaki, 2024). They raised concerns about our statistical analysis, the discrepant findings between previous studies and ours, and the potential influence of single‐ versus multiple‐set protocols on these discrepancies.

To address the statistical issue, we have re‐analysed the relationship between the total exercise volume and the percentage change in *R*_max_ (the number of repetitions achieved during elbow flexions at 30% of one‐repetition maximum) using a linear mixed model (R software, v.4.4.0) with the restricted maximum likelihood method (Cnaan et al., 1997). This approach accounts for the random effects of repeated measures (i.e., the left and right arms) from the same participants while testing the fixed effect of interest. The analysis revealed a significant effect of total exercise volume on the percentage change in *R*_max_ (*t* = 8.912, *P* < 0.001), indicating a strong positive relationship between the two variables. The model estimate revealed that for every 1000 kg × repetitions increase in exercise volume, the percentage change in *R*_max_ is expected to rise by 41.0% ± 4.6%. These results suggest that our original conclusion was valid regardless of whether the assumption of independence was violated.

We acknowledge that some studies cited in their letter (Buckner et al., 2020; Fahs et al., 2015; Jessee et al., 2018) have suggested the benefits of blood flow restriction (BFR) on muscle endurance adaptations, considering lower exercise volumes in BFR than in free blood flow (FBF). However, we expect that the differences in exercise progression and the magnitude of improvement in muscle endurance (particularly in FBF conditions) are major contributors to the discrepancies in results between these studies and ours.

Fahs et al. (2015) used an exercise protocol in which the cuff pressure, the number of sets and the absolute load were increased during 6‐week training period. Their strategy clearly differed from ours, which aimed to maximize the exercise duration while maintaining a constant absolute load. Considering the principle of training specificity, their protocol should be less optimal for increasing the number of repetitions than ours. In fact, there was a considerable difference in the improvement in muscle endurance, despite the larger number of exercise sessions in their study (18) than in ours (12). From table 2 of Fahs et al. (2015), we calculated the pretraining to post‐training difference in means (note that this value is somewhat different from the mean pretraining to post‐training difference), finding ∼30% and ∼40% improvements in muscle endurance in the BFR and FBF (abbreviated as FF in their study) limbs, respectively. These values were much smaller than ours (66.8% and 287.5% improvements in the BFR and FBF, respectively), even when considering the differences in muscle group, load adjustment and cadence of the endurance test.

Jessee et al. (2018) and Buckner et al. (2020) studied different muscle groups but used the same protocol that limits the number of repetitions to 90 per set. As Buckner et al. (2020) report, even at the midpoint of their training period, only 31% of participants in the FBF (abbreviated as 1500 in their study) but 95% and 52% of participants in the high‐ and moderate‐pressure BFR, respectively, reached volitional failure by the fourth set. These data imply that their FBF protocol has a limitation similar to that of repetition‐matched control commonly used in previous studies on BFR training, where the effort level remains submaximal. Limiting the number of repetitions and the effort level will undoubtedly compromise the muscle adaptations to exercise.

Given the differences in exercise progression and improvement in muscle endurance, it is not surprising that our results disagree with those of the above‐mentioned studies. Our results are relatively consistent with those of Farup et al. (2015), who used a multiple‐set protocol (four sets to failure at 40% of one‐repetition maximum) for elbow flexion exercise. According to their fig. 2, the number of repetitions in the first set averaged over a 6‐week training period was nearly 50 and 150 in the BFR and FBF conditions, respectively. These values and the impact of BFR are comparable to those observed in our study (71.4 and 195.0 in the BFR and FBF, respectively). Although Farup et al. (2015) did not report the training‐induced change in muscle endurance, the similar effect of BFR on exercise volume between their study and ours suggests that the discrepancies in findings noted by Hammert et al. (2024) might not stem from the use of single‐ versus multiple‐set protocols.

From a physiological point of view, the exercises optimal for improving muscle endurance cannot be the same as those optimal for muscle hypertrophy. We thus believe that the BFR‐induced reduction in the improvement in muscle endurance during submaximal contractions can be viewed as a reflection of the positive effects on muscle hypertrophy and anaerobic metabolism. Furthermore, as mentioned in the introduction of our paper (Ida & Sasaki, 2024), the term ‘muscle endurance’ has a broad meaning. Indeed, we found no significant difference in the improvements in ‘half‐time’, another indicator of muscle endurance, between the BFR and FBF arms. Considering the low exercise volume in BFR compared with FBF, we speculate that the application of BFR might have some effect on improving the capacity to maintain maximal force production. To date, BFR exercises have been developed in a way that optimizes muscle hypertrophy and gain in strength rather than an improvement in endurance. We agree with Hammert et al. (2024) and with Dr Wernbom's viewpoint (Wernbom, 2024) that future research is warranted to clarify the mechanisms determining and improving muscle endurance using this interesting exercise model.

## AUTHOR CONTRIBUTIONS

Both authors have approved the final version of the manuscript and agree to be accountable for all aspects of the work. Both persons designated as authors qualify for authorship, and all those who qualify for authorship are listed.

## CONFLICT OF INTEREST

The authors declare no conflicts of interest.

## FUNDING INFORMATION

None.
